# Identification of a Reliable Biomarker Profile for the Diagnosis of Gaucher Disease Type 1 Patients Using a Mass Spectrometry-Based Metabolomic Approach

**DOI:** 10.3390/ijms21217869

**Published:** 2020-10-23

**Authors:** Iskren Menkovic, Michel Boutin, Abdulfatah Alayoubi, François E. Mercier, Georges-Étienne Rivard, Christiane Auray-Blais

**Affiliations:** 1 Division of Medical Genetics, Department of Pediatrics, Centre de Recherche-CHUS, Faculty of Medicine and Health Sciences, Université de Sherbrooke, CIUSSS de l’Estrie-CHUS, 3001, 12th Avenue North, Sherbrooke, QC J1H 5N4, Canada; iskren.menkovic@usherbrooke.ca (I.M.); michel.boutin2@usherbrooke.ca (M.B.); 2 Divisions of Experimental Medicine and Hematology, Department of Medicine, Faculty of Medicine, McGill University, Lady Davis Institute for Medical Research, Jewish General Hospital, 3755, Côte Sainte-Catherine, Montreal, QC H3T 1E2, Canada; abdulfatah.alayoubi@mail.mcgill.ca (A.A.); francois.mercier@mcgill.ca (F.E.M.); 3 Department of Biochemistry and Molecular Medicine, College of Medicine, Taibah University, University Road, Madinah 42353, Saudi Arabia; 4 Division of Hemato-Oncology, Department of Pediatrics, Faculty of Medicine, Centre Hospitalier Universitaire Sainte-Justine, 3175, Côte Sainte-Catherine, Montreal, QC H3T 1C5, Canada; georges-etienne.rivard.hsj@ssss.gouv.qc.ca

**Keywords:** Gaucher disease, metabolomics, mass spectrometry, glucosylsphingosine (lyso-Gb_1_), lyso-Gb_1_ analogs, N-palmitoyl-O-phosphocholineserine, sphingosylphosphorylcholine, biomarkers, plasma

## Abstract

Gaucher disease (GD) is a rare autosomal recessive multisystemic lysosomal storage disorder presenting a marked phenotypic and genotypic variability. GD is caused by a deficiency in the glucocerebrosidase enzyme. The diagnosis of GD remains challenging because of the large clinical spectrum associated with the disease. Moreover, GD biomarkers are often not sensitive enough and can be subject to polymorphic variations. The main objective of this study was to perform a metabolomic study using an ultra-performance liquid chromatography system coupled to a time-of-flight mass spectrometer to identify novel GD biomarkers. Following the analysis of plasma samples from patients with GD, and age- and gender-matched control samples, supervised statistical analyses were used to find the best molecules to differentiate the two groups. Targeted biomarkers were structurally elucidated using accurate mass measurements and tandem mass spectrometry. This metabolomic study was successful in highlighting seven biomarkers associated with GD. Fragmentation tests revealed that these latter biomarkers were lyso-Gb_1_ (glucosylsphingosine) and four related analogs (with the following modifications on the sphingosine moiety: -C_2_H_4_, -H_2_, -H_2_+O, and +H_2_O), sphingosylphosphorylcholine, and N-palmitoyl-O-phosphocholineserine. Based on the plasma biomarker distribution, we suggest the evaluation of this GD biomarker profile, which might facilitate early diagnosis, monitoring, and follow-up of patients.

## 1. Introduction

Lysosomal storage disorders (LSDs) are rare inherited metabolic conditions characterized by the inability of the lysosomes to process substrate(s), resulting in the accumulation of these compounds within the lysosome and ultimately leading to progressive and multisystemic clinical manifestations. Gaucher Disease type 1 (GD; OMIM 230800) is the most prevalent LSD with approximately 1 case in 40,000 births in the general population and 1 case in 800 births in the Ashkenazi Jewish population [1]. GD is the result of biallelic mutations in the *GBA* gene (1q22) leading to a deficiency of glucocerebrosidase, an enzyme involved in the catabolism of glucosylceramide (Gb_1_) [1,2]. This enzymatic defect leads to the accumulation of Gb_1_ in the lysosome of reticuloendothelial cells, ultimately resulting in the formation of Gaucher cells [3,4]. Cytopenia, hepatosplenomegaly, and bone lesions are usually observed in GD patients due to Gaucher cell’s infiltration of the liver, spleen, and bone marrow [4,5]. From a clinical perspective, three subtypes of GD can be observed based on the absence, presence, and severity of neurological involvement. In Gaucher disease type 1, which is the most common form of the disease, representing 90–95% of all GD cases, patients may, in some cases, experience milder neurological impairments, whereas in GD types 2 and 3, patients experience more severe symptoms, such as abnormal eye movements and seizures, along with brain damage [6,7]. Type 2 Gaucher disease is usually observed in infancy, whereas type 3 GD is detected in childhood and tends to progress more slowly than GD type 2 [8]. However, it is worth noting that there are no clear distinctions between the type 2 and type 3 subtypes of GD but rather a phenotypic continuum ranging from horizontal ophthalmoplegia as the only neurological involvement in GD type 3 to hydrops fetalis in severe cases of GD type 2 [5,9,10].

While enzyme replacement therapy (ERT) is the most frequently used treatment, substrate reduction therapy (SRT) may also be offered to patients [11]. While these treatments are effective to different extents, studies have demonstrated that early initiation of treatment, when required, will ultimately result in better outcomes for the patients [11]. However, due to the large clinical spectrum and heterogeneity of the disease, early detection and confirmation of the diagnosis of GD patients remain challenging, thus highlighting the need for reliable GD biomarkers. Biomarkers, such as C-C motif chemokine ligand (CCL18/PARC), angiotensin-converting enzyme (ACE), tartrate-resistant acid phosphatase 5b (TRAP5b), and C-terminal telopeptide of type 1 collagen (CTx), may lead to the diagnosis of a GD case, but the quantitation of these compounds generally lack sensitivity and most of them are not specific to GD [12,13,14,15,16]. Chitotriosidase, an enzyme produced by activated macrophages, can be an interesting biomarker for the monitoring and follow-up of patients. However, it is not a reliable biomarker for the diagnosis of the disease, due to the high levels of polymorphism present in about 6% of the general population. This leads to major differences in baseline levels among GD patients, where some do not express chitotriosidase at all [17].

More recently, glucosylsphingosine (lyso-Gb_1_), a substrate generated following the deacylation of glucosylceramide by lysosomal acid ceramidase [18], has been observed in the plasma of GD patients and has since gained interest in the medical and scientific community [19,20,21]. Unlike most of the previously mentioned biomarkers, lyso-Gb_1_ is specific to GD and is potentially involved in the pathogenesis of the disease, more specifically regarding the immune system, as well as the skeletal and neurological systems [22]. Studies also tend to demonstrate that levels of lyso-Gb_1_ can be useful biomarkers for the diagnostic, prognostic, monitoring, and follow up of GD patients [23]. Previous studies revealed that lyso-Gb_1_ in matrices such as dried blood spot or plasma can be reliable biomarkers for treatment efficiency and follow-ups of treated and untreated GD patients [24]. In fact, lyso-Gb_1_ has been evaluated for a median range of 22 years as part of a long-term follow up of a large cohort of GD patients [25]. While lyso-Gb_1_ appears to be a promising biomarker, we believe, based on research previously done by our group, that there might be other interesting biomarkers for GD patients that reflect the severity and progression of the disease in all GD patients. Indeed, previous metabolomic studies performed for Fabry disease, another lysosomal storage disorder, revealed that globotriaosylsphingosine (lyso-Gb_3_) is not a unique biomarker for Fabry disease patients. Our studies showed that analogs of lyso-Gb_3_, which are lyso-Gb_3_ molecules with modified sphingosine moieties, are present in both plasma and urine of Fabry patients and are relevant to the disease [26,27,28,29]. We therefore suggest that a similar GD pattern may occur, since both GD and Fabry disease are involved in the same metabolic pathway. Mirzain et al., hypothesized in 2015 that the chemical modifications observed on lyso-Gb_3_ to generate the analogs in Fabry disease (which were discovered by Auray-Blais et al.) may also be present on lyso-Gb_1_ to generate the analogs in Gaucher disease [30]. Their hypothesis proved to be correct, and this led to the identification and quantification of some lyso-Gb_1_ analogs in GD plasma and urine specimens [30]. However, the compounds quantified by Mirzaian et al. were not characterized by mass spectrometry as being lyso-Gb_1_ analogs, since no fragmentation studies were performed. Moreover, the chromatographic method proposed by Mirzaian et al. was not able to separate lyso-Gb_1_ from galactosylsphingosine (psychosine), the latter being a biomarker for Krabbe disease [31]. Furthermore, compounds quantified by Mirzaian et al. were not subject to prior metabolomic studies, leading us to believe that other insightful molecules might be reliable biomarkers for GD.

The main objective of this study was to perform a semi-targeted plasma metabolomic study using ultra-performance liquid chromatography (UPLC) coupled to time-of-flight (TOF) mass spectrometry (MS) in order to identify a profile of sensitive and specific GD biomarkers that could be used for early detection, prognosis, monitoring, and follow up of affected patients. The secondary objective targeted the structural elucidation of biomarkers using accurate mass measurements and tandem mass spectrometry.

## 2. Results and Discussion

**Figure 1:**
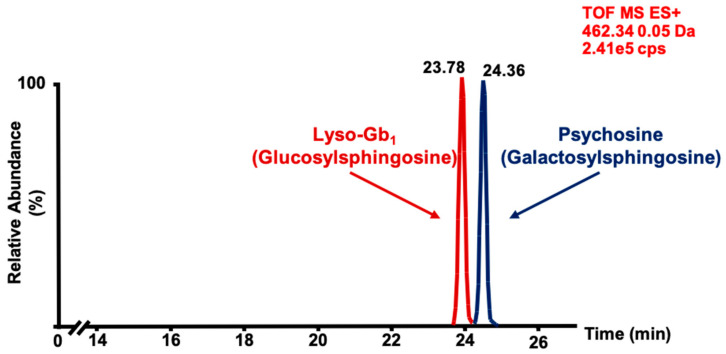
Chromatographic separation of glucosylsphingosine (lyso-Gb_1_; 5 μg on column, retention time: 23.78 min) and psychosine (5 μg on column, retention time: 24.36 min) commercial standards analyzed by time-of-flight mass spectrometry (MS-TOF) with a mass window of 0.05 Da. Peaks associated with each compound were confirmed by individual analysis of each standard. ES+ = positive electrospray.

Before the development of the chromatographic separation method, we were aware of the existence of psychosine, a biomarker increased in Krabbe disease [32] which might interfere with the quantitation of lyso-Gb_1_ considering that it is a molecule with physical and chemical properties similar to lyso-Gb_1_ [33]. Therefore, in order to properly characterize the compound identified and better understand the physiopathology of Gaucher disease, a separation of psychosine and lyso-Gb_1_ is preferred. Since the two compounds are differentiated only by the conformation of one hydroxyl group, normal phase chromatography was chosen for their separation, as shown in Figure 1. Based on the retention times of these two compounds, it was possible to extrapolate the retention times of the lyso-Gb_1_ analogs based on their relative polarity, hence confirming that the analogs analyzed were from lyso-Gb_1_ and not psychosine.

**Figure 2:**
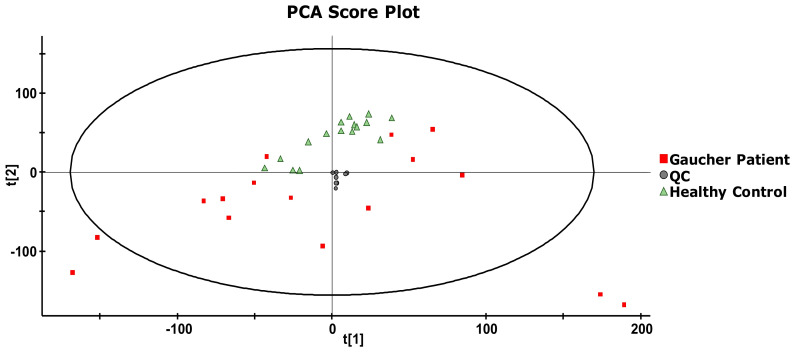
A principal component analysis (PCA) score plot derived from the metabolomic analysis of plasma samples from untreated Gaucher disease (GD) patients (*n* = 16), age- and gender-matched healthy controls (*n* = 16), and quality control (QC) replicate injections (*n* = 8). The ellipse corresponds to the Hotelling T2 range with a significance level of *p* = 0.05.

Data mining steps with the use of MarkerLynx (version 4.1), following the analysis of 16 untreated GD patients and 16 age- and gender-matched healthy controls, yielded a total of 91,197 markers, which were aligned based on the retention time and accurate mass. Data were then imported in EZinfo for further multivariate statistical analyses. First, the system stability was evaluated using principal component analysis (PCA). Briefly, the PCA score plot allowed an evaluation of how similar or different samples were in terms of compositions according to their respective position on the score plot. Based on their chemical compositions, the more similar two samples were, the closer they appeared on the PCA score plot. Considering that quality controls (QCs) were replicate injections of a single sample, it was expected that QCs should be closely positioned on the PCA score plot. As shown in Figure 2, QCs (*n* = 8) (represented in red) were indeed very close, confirming that the system, as well as the samples were stable for the entire duration of the study. This also indicates that variations observed in metabolite concentrations in the samples analyzed were not system-related but rather due to biological reasons. Proximity in the location of QCs also indicates that data alignment parameters were adequate for the study. Moreover, considering that QCs were obtained by sampling and mixing equal volume (20 μL) from each healthy control and GD patient sample, it was expected that QCs should be located in the center of the PCA score plot, as shown in Figure 2, also confirming the reliability of the analyses done during this study.

**Figure 3:**
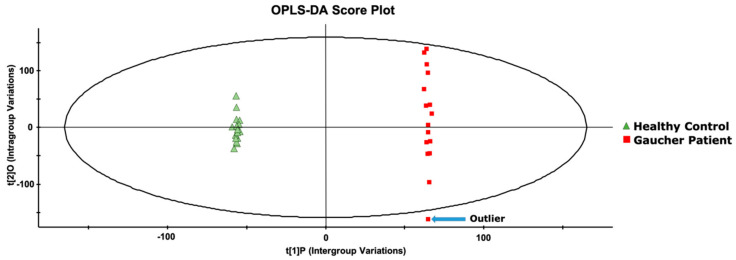
An orthogonal partial least-square discriminant analysis (OPLS-DA) score plot, resulting from the metabolomic analysis of plasma samples from untreated GD patients (*n* = 16) and age- and gender-matched healthy controls (*n* = 16). The ellipse corresponds to the Hotelling T2 range with a significance level of *p* = 0.05.

Once the system stability was confirmed, an orthogonal partial least-square discriminant analysis (OPLS-DA) was performed to identify compounds that would allow to separate the two groups under investigation. As shown in Figure 3, complete segregation of GD patients’ samples and healthy control samples was achieved. It is important to note that based on the OPLS-DA chart, one patient was considered an outlier since he was plotted outside the Hotelling T2 range with a significance level of *p* = 0.05. This patient was affected with a mild form of GD type 1, which led to the system qualifying him as an outlier. However, he was not excluded from this study.

**Figure 4:**
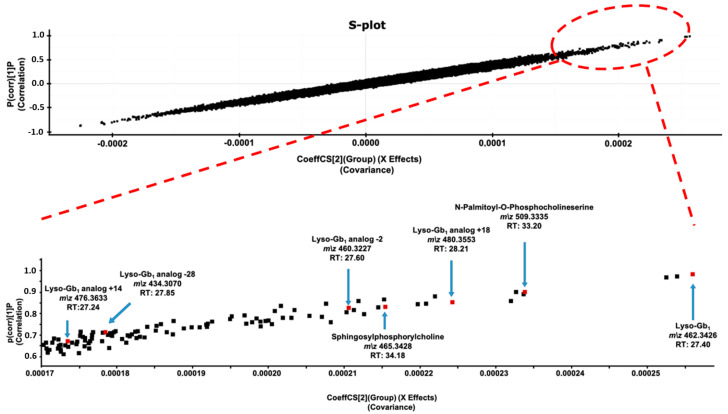
S-plot showing the correlation in function of the covariance for the metabolites used to segregate GD patients and age- and gender-matched healthy controls in the OPLS-DA score plot. Specific positions of biomarkers (lyso-Gb_1_; lyso-Gb_1_ analogs −28 Da, −2 Da, +14 Da, and +18 Da; N-palmitoyl-O-phosphocholineserine; and sphingosylphosphorylcholine) are zoomed in the lower part of the figure. Exact positions are indicated by a red square and an arrow. Accurate mass measured, as well as their respective retention times (RTs) in min, are indicated for each biomarker.

**Figure A1:**
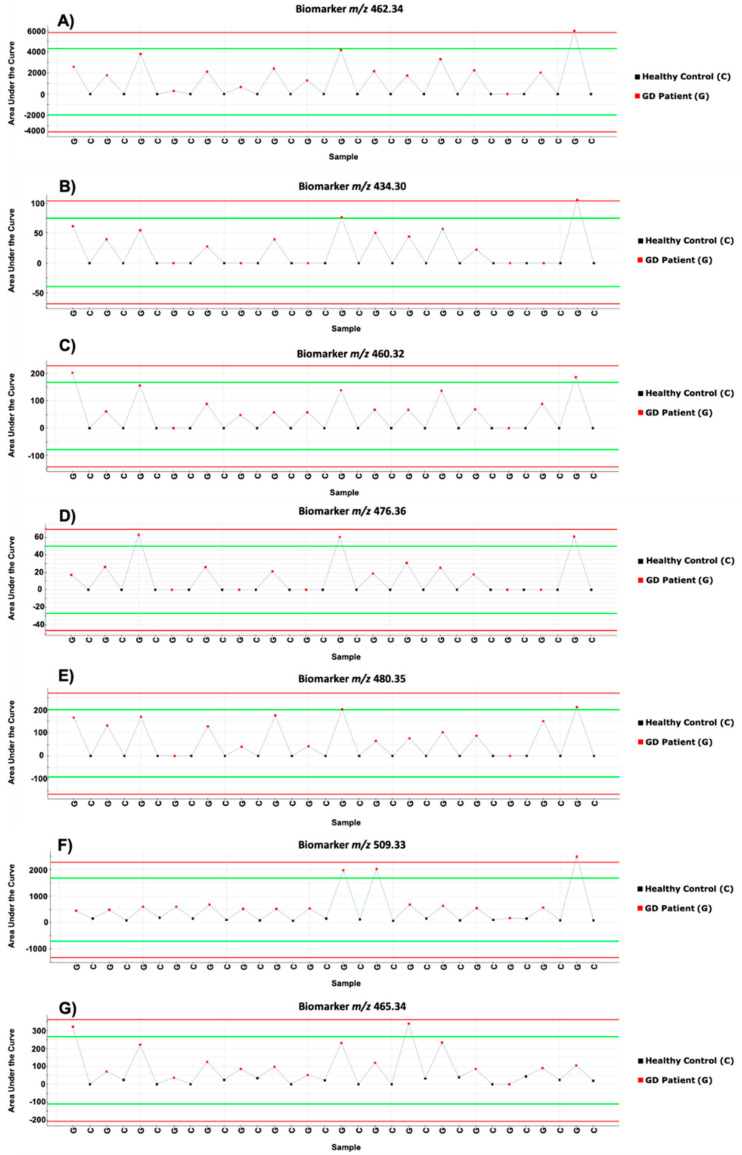
Trend plot charts for lyso-Gb_1_ (**A**), lyso-Gb_1_ analogs −28 Da (**B**), −2 Da (**C**), + 14 Da (**D**), +18 Da (**E**), N-palmitoyl-O-phosphocholineserine (**F**), and sphingosylphosphorylcholine (**G**). G = Gaucher patient, C = healthy control.

The metabolites which contributed most to the segregation of the two sample groups on the OPLS-DA score plot were detected using an S-plot (Figure 4). The more a compound was abundant in GD samples compared to healthy control samples, further it was positioned in the upper right-side corner of the graph. Conversely, the more a compound was abundant in healthy control samples compared to GD samples, further it was positioned in the lower left-side corner of the graph. Metabolites showing the highest separation power were located at the extremities of the S-plot graph. While both sides of the graph were investigated to identify potential biomarkers for GD using trend plot charts (Figure A1), as well as a visual inspection of the chromatographic peak associated with the compound, only some metabolites located in the upper right-side corner of the graph ultimately resulted in potential GD biomarkers. Markers observed in less than 50% of GD samples, or that were present only in GD samples collected from one hospital or detected at higher concentration in one or some control samples, compared to GD samples, were automatically discarded. Ultimately, 7 compounds with mass to charge ratios (*m/z*) of 434.3074, 460.3227, 462.3426, 465.3428, 476.3633, 480.3553, and 509.3335 were selected as potential GD biomarkers and further investigated using fragmentation tests to identify their structures (Figure 4).

To ensure that fragmentation tests would produce fragments of sufficient intensity to obtain their accurate mass, samples were further concentrated. While sample purification remained the same, samples were resuspended in 100 μL instead of 250 μL to provide a concentration factor of 2.5 for structural elucidation of all potential biomarkers, except analogs −24 and +14, which were further concentrated five times to obtain a concentration factor of 12.5. Based on its *m/z*, the compound *m/z* 462.3426 was suspected to be lyso-Gb_1_, which was later confirmed based on the fragmentation pattern. It was also observed that metabolites *m/z* 434.3074, 476.3633, 480.3553, and 460.3227 had very similar fragmentation patterns to lyso-Gb_1_, suggesting that these four metabolites are analogs of lyso-Gb_1_. Based on the mass difference from lyso-Gb_1_, it is believed that these four potential biomarkers are, in fact, lyso-Gb_1_, with an extra double bond, which accounts for the loss of 2 H (−2 Da) for metabolite *m/z* 460.3227, a loss of C_2_H_4_ (−28 Da) for metabolite *m/z* 434.3074, and a loss of 2 H but a gain of O (+14 Da) for metabolite *m/z* 476.3633, and, finally, metabolite *m/z* 480.3553 possesses a hydrated sphingosine instead of a double-bond, which accounts for the gain of 18 Da. According to the fragmentation spectra obtained, all those proposed structural modifications were located on the sphingosine. In a recent study by Mirzaian et al. [30], the authors previously identified and quantified the following lyso-Gb_1_ analogs −28 Da, −2 Da, and +14 Da, which were also highlighted in this study. However, the lyso-Gb_1_ analog at +18 Da has never been described in the literature.

**Figure 5:**
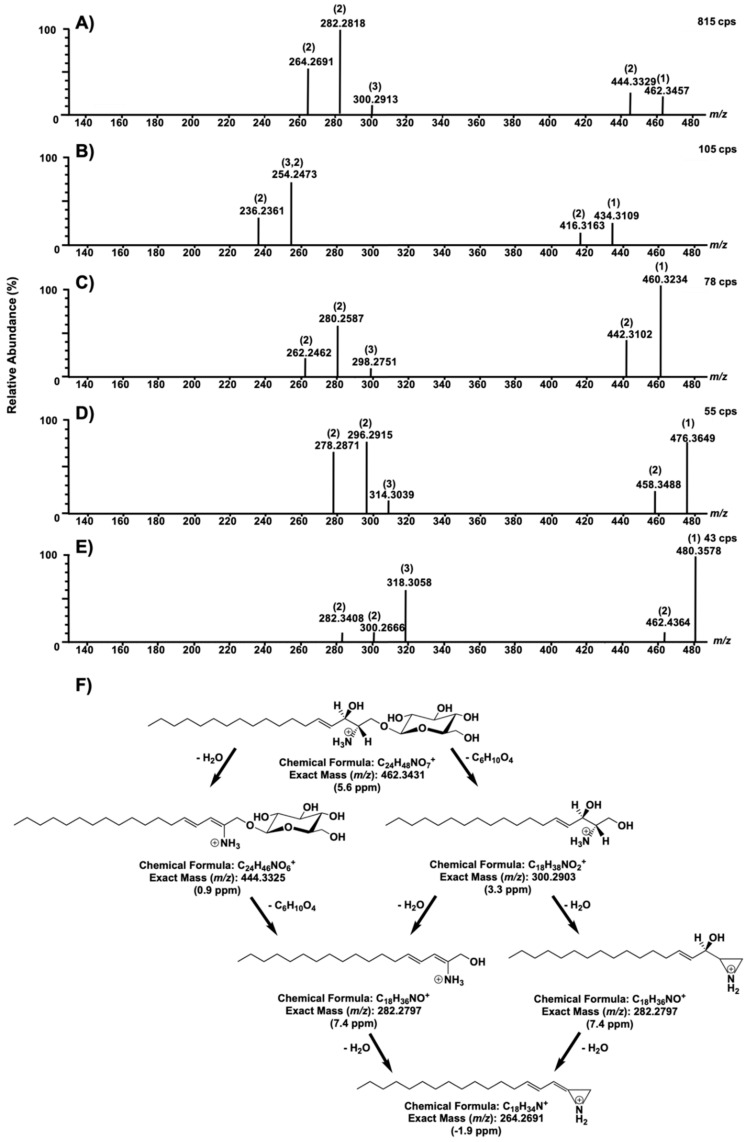
Fragmentation test results for compounds with *m/z* 462.3426 (**A**), 460.3227 (**B**), 480.3553 (**C**), 434.3074 (**D**), and 76.3633 (**E**), with a collision energy ramp of 10–30 V. In each spectrum, (1) corresponds to the precursor ion, (2) indicates a loss of a water molecule from the previous fragment, and (3) represents a loss of a molecule of glucose. A fragmentation mechanism for lyso-Gb_1_ (*m/z* 462.3227) is proposed in (**F**).

The fragmentation pattern for each molecule and a general fragmentation mechanism for lyso-Gb_1_ is shown in Figure 5.

**Figure 6:**
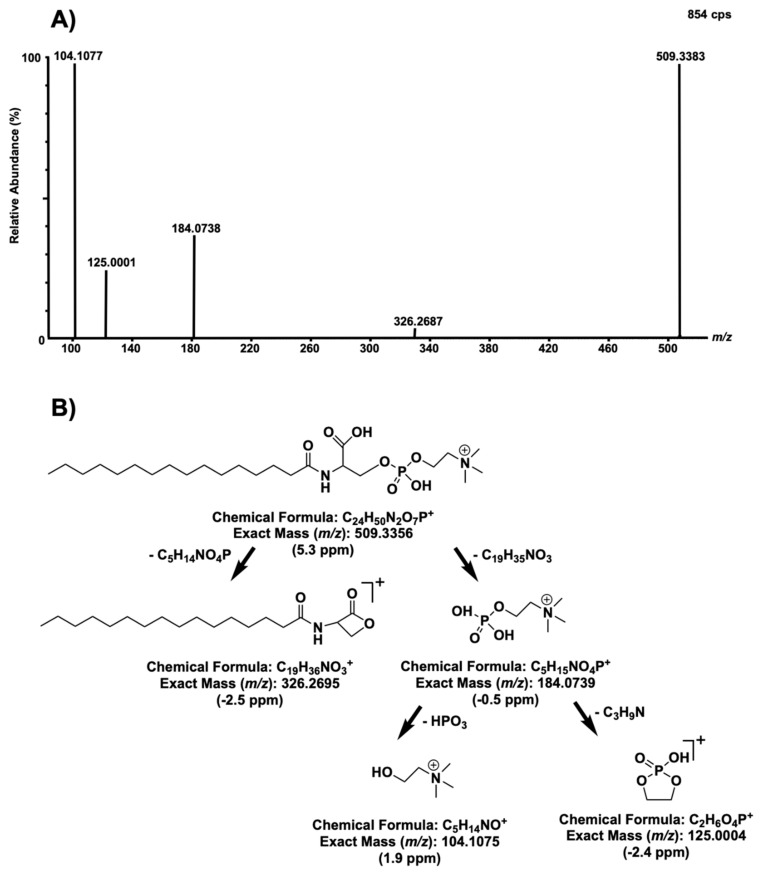
Fragmentation tests of compounds with *m/z* 509 with a collision energy ramp of 10–25 V (**A**) and a fragmentation mechanism previously proposed by Sidhu et al. [30], with theoretical masses and measured mass errors in part per million (ppm) (**B**).

Following fragmentation tests, it was observed for biomarkers *m/z* 465.3428 and 509.3335 that a phosphocholine (PC) head group was present in both of these compounds based on the typical fragmentation pattern of PC heads, resulting in fragments with *m/z* at 184.072, 125.000, and 104.110. However, excluding the PC head, the two aforementioned molecules had different fragmentation patterns, suggesting that they may not be as closely structurally related. Fragments from biomarker *m/z* 509 confirmed that it is a biomarker previously known as lyso-sphingomyelin-509 (lysoSM-509) (Figure 6).

Recently, Sidhu et al. proposed a structure for lysoSM-509 based on products obtained following precise chemical derivatization, aimed at confirming the presence of specific functional groups [34]. Ultimately, Sidhu et al. suggested that lysoSM-509 could be N-palmitoyl-O-phosphocholineserine. Fragments of the previously mentioned compound matched fragments observed in this study, confirming that the biomarker at *m/z* 509.3383 is N-palmitoyl-O-phosphocholineserine. However, Sidhu et al. also observed N-palmitoyl-O-phosphocholineserine in Niemann–Pick disease patients, suggesting that this latter biomarker is not a specific biomarker for Gaucher disease but rather an indicator of a sphingolipidosis [34,35].

**Figure 7:**
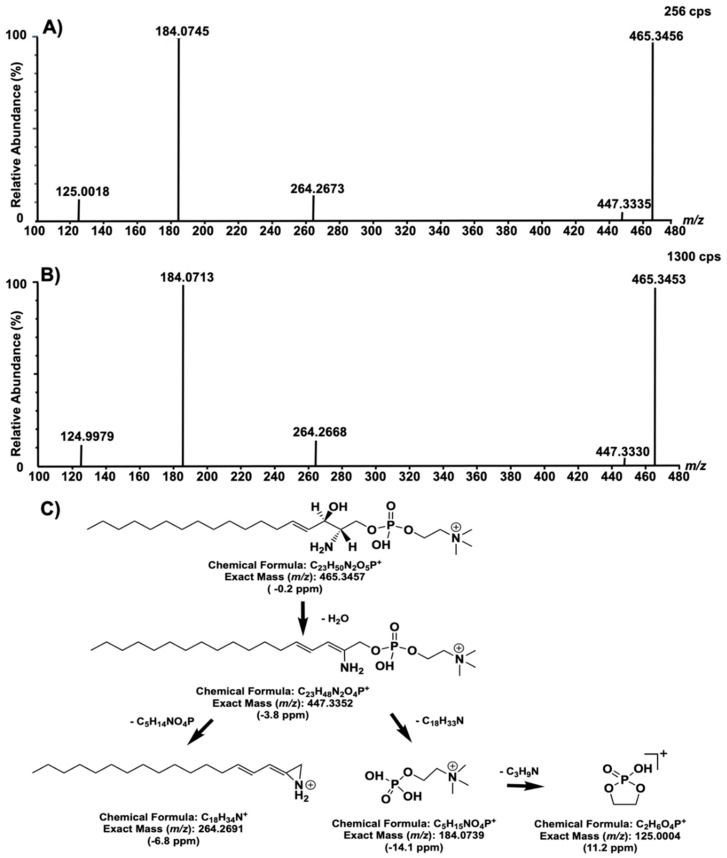
Fragmentation test results of compounds with *m/z* 465, with a collision energy ramp of 15–25 V (**A**). Fragmentation test results of a standard of sphingosylphosphorylcholine (d18:1) (10 μg on column) (**B**) and a proposed fragmentation mechanism with theoretical and experimental exact mass differences (**C**).

Finally, the biomarker at *m/z* 465 was structurally elucidated based on the accurate mass measured and the fragmentation patterns (Figure 7). The proposed structure was ultimately confirmed by comparing fragments and their relative abundance, as well as the retention time of biomarker *m/z* 465 with fragments of a standard of sphingosylphosphorylcholine (SPC) (d18:1).

**Figure 8:**
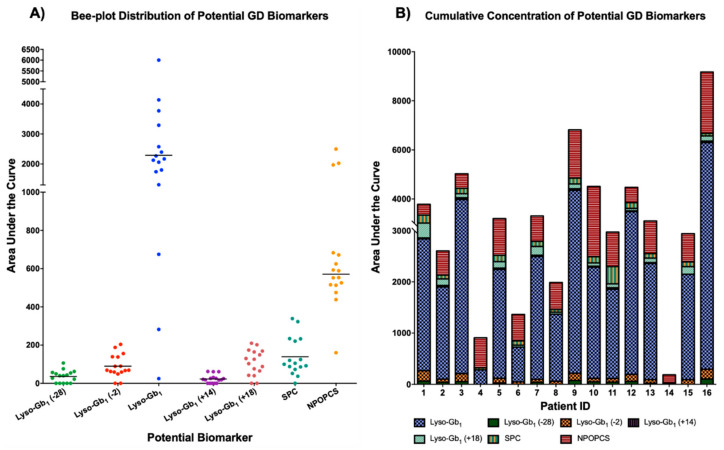
Bee-plot distribution (**A**) and cumulative concentrations (**B**) of potential GD biomarkers highlighted in the current metabolomic study. Sphingosylphosphorylcholine (SPC) and N-palmitoyl-O-phosphocholineserine (NPOPCS). The line in (**A**) represents the median value for each potential biomarker. All potential GD biomarkers were not observed in healthy controls.

Studies have demonstrated that SPC is a constituent of lipoproteins and a potentially important lipid mediator of cell type-specific functions in certain tissues. In fact, Thomas et al., have shown evidence that SPC in rats “is a powerful vasoconstrictor of intrapulmonary arteries (IPA) and the novel finding that SPC-induced vasoconstriction in IPA is dependent on activation of a Ca^2+^” is important, since it can mediate certain cell mechanisms, such as contractility and cell proliferation [36]. Moreover, SPC may also be implicated in chronic vascular disease with involvement in processes such as proliferation and migration of vascular smooth muscle cells by acting as a pro-inflammatory mediator by activating p38 mitogen-activated protein kinases [36]. This is consistent with inflammation being involved in the pathogenesis of GD [37]. However, SPC (similar to N-palmitoyl-O-phosphocholineserine) is not specific to Gaucher disease. Indeed, significantly increased concentrations of SPC were observed in patients with acid sphingomyelinase deficiency such as Niemann–Pick type A and type B [38,39]. However, this metabolomic study showed the importance of SPC quantitation, even in GD patients considering the segregation power of the biomarker based on its position in the S-plot graph (Figure 4). More specifically, the increase of each potential biomarker identified during this metabolomic study is shown in Figure 8 for each GD patient. These potential biomarkers were not detected in healthy control samples.

**Figure A2:**
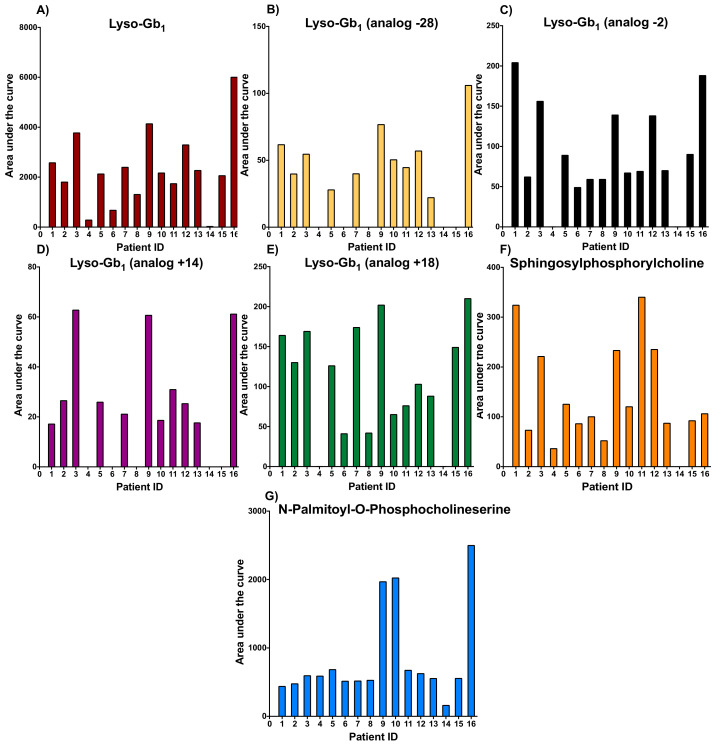
Areas under the curve are shown for lyso-Gb_1_ (**A**), lyso-Gb_1_ analog +18 Da (**B**), lyso-Gb_1_ analog −2 Da (**C**), lyso-Gb_1_ analog −28 Da (**D**), lyso-Gb_1_ analog +14 Da (**E**), sphingosylphosphorylcholine (**F**), and N-palmitoyl-O-phosphocholineserine (**G**). These biomarkers were not detected in control samples.

Our results show that N-palmitoyl-O-phosphocholineserine (G in Figure A2) was the only marker to be elevated in all GD samples. An increase in the concentration of lyso-Gb_1_, a biomarker known for its diagnostic utility, as well as its monitoring performance in GD, was observed in all samples analyzed, except for one patient (ID 14), who was affected with a more attenuated form of the disease. [40,41]. While N-palmitoyl-O-phosphocholineserine seems to be a sensitive biomarker for GD, it is not specific to the disease, but is rather an indicator of a sphingolipidosis. N-palmitoyl-O-phosphocholineserine is also a biomarker for Niemann–Pick type C disease, while SPC is a biomarker for Niemann–Pick type A and type B diseases [38,39,42]. Therefore, a diagnostic of GD only based on that compound would not be possible.

Additionally, lyso-Gb_1_ was significantly more abundant than all the other analogs in plasma. In terms of distribution, lyso-Gb_1_ represents over 91.4% of the total lyso-Gb_1_ content, including the analogs. Meanwhile, the analogs at −28 Da, −2 Da, +14 Da, and +18 Da represent approximately 1.6%, 2.9%, 0.9%, and 3.2% of the total lyso-Gb_1_ + analogs, respectively. The recovery of lyso-Gb_1_ after solid phase extraction (SPE) was evaluated using a plasma sample spiked with 20 μg of lyso-Gb_1_ commercial standard, either pre (*n* = 3) or post-SPE (*n* = 3) purification. The recovery rate for lyso-Gb_1_ was calculated to be over 92%, thus suggesting that sample preparation was not a major source of lyso-Gb_1_ analog loss. While lyso-Gb_1_ seems to be a more abundant biomarker than its analogs in plasma for GD patients, we strongly believe that lyso-Gb_1_ and its related analogs should all be evaluated and quantified, since these analogs may correlate with certain specific clinical manifestations in GD, as found in Fabry disease [43]. There seems to be a correlation between the bone marrow burden (BMB) score observed by MRI and the levels of lyso-Gb_1_ and its analogs. Moreover, for some patients, the levels of the analog at −2 Da were higher than those measured for the analog +18 Da, while, for other patients, it was the opposite. Regarding SPC, an elevation of the biomarker was observed in all patients, except patient ID 14, who presented a less severe form of the disease and lower levels of lyso-Gb_1_ (see Table 1, Figure A2). Unlike lyso-Gb_1_ and its analogs, as well as N-palmitoyl-O-phosphocholineserine, increased levels of SPC did not correlate with a more severe form of the disease. In fact, GD patient ID 16 had a particularly high BMD score, as shown in Table 1, compared to all other GD patients, yet his SPC levels were not significantly more elevated than other patients. Other compounds, such as glucosylceramide, a substrate normally catabolized by the enzyme glucocerebrosidase, which is deficient in GD patients, may potentially be an interesting biomarker for the disease. However, in order to investigate the relevancy of glucosylceramide and other similar compounds, a different sample preparation approach will be required.

## 3. Materials and Methods

### 3.1. Ethics Approval

This project was approved by the Research Ethics Board (REB) of the Faculty of Medicine and Health Sciences of the Centre Hospitalier Universitaire de Sherbrooke (CHUS: Project ID MP-31-2017-1414, 28th September 2016), as well as the REB of Montreal’s Jewish General Hospital (Project ID MEO-31-2020-1937, 22nd November 2019) and Centre Hospitalier Universitaire Sainte-Justine as a multicentric evaluation (Project ID MEO-31-2021-2782, 28th September 2016). Informed consent from all GD patients and healthy controls were obtained.

### 3.2. Patients and Controls

Blood samples were collected from 16 untreated patients previously diagnosed with GD (age range: 23–68 years old; 8 males, 8 females) and 16 age- and gender-matched controls (age range: 26–66 years old; 9 males, 7 females). Adult men and women with a confirmed diagnosis of GD type 1 were included in this study. Treated GD type 1 patients, as well all GD type 2 and type 3 patients, were excluded from this study. Controls recruited did not have any LSDs or other comorbidities. The diagnosis was confirmed for each GD patient by demonstrating marked enzyme deficiency of β-glucosidase in peripheral blood leukocytes and sequencing of the *GBA* gene. Only untreated GD patient samples were considered for this metabolomic study to ensure that a significant increase of potential GD biomarkers would be observed.

#### Clinical Features and Biochemical Parameters

GD patients’ demographics, mutations, treatment, and signs and symptoms are shown in Table 1. Some patients had comorbidities, such as diabetes (patients 4 and 11), ovarian adenocarcinoma (patients 8 and 13), and hypercholesterolemia (patients 4 and 10). Patient 6 had unconjugated hyperbilirubinemia, while patient 9 was suffering from minor beta thalassemia and multiple cystic lesions in the liver and spleen.

### 3.3. Whole Blood and Plasma Specimen Collection and Processing

Using a 21 G butterfly needle, whole blood samples were collected into BD Vacutainer tubes with sodium citrate as an anticoagulant. Plasma was immediately extracted by centrifugation at 2000 g for 10 min and transferred into 15 mL Falcon tubes. Plasma samples were stored at −80 °C until the metabolomic study was performed.

### 3.4. Reagents

Glucosysphingosine (lyso-Gb_1_; >98%) and galactosylsphingosine (Psychosine; >98%) were purchased from Matreya (Pleasant Gap, PA, USA). LC-MS grade acetonitrile (ACN) and HPLC grade methanol (MeOH) were purchased from EMD Chemicals Inc. (Darmstadt, Germany), while Optima LC-MS grade water, American Clinical Society (ACS) grade ammonium formate (Amm. Form.), o-phosphoric acid (H_3_PO_4_; 85%), and ammonium hydroxide (NH_4_OH; 28%–30% purity) were obtained from Fisher Scientific (Fair Lawn, NJ, USA). ReagentPlus grade dimethyl sulfoxide (DMSO; ≥99.5%) and terfenadine were obtained from Sigma–Aldrich (St. Louis, MO, USA), and, finally, formic acid (FA; >99%) was purchased from Acros Organics (Morris Plain, NJ, USA).

### 3.5. Sample Preparation

Untreated GD patients or healthy control plasma samples were vortexed for 10 s, and 200 μL of plasma was then transferred into 2 mL Eppendorf plastic tubes. A total of 500 μL of H_3_PO_4_ (2% in water) and 500 μL of MeOH were added to the plasma sample, followed by 10 s of mixing (vortex). Sample mixtures were transferred to mixed-mode cation-exchange cartridges (Oasis MCX, 30 mg, 60 μm, Waters Corp., Milford, MA, USA), previously conditioned with 1000 μL of MeOH and 1000 μL of 2% H_3_PO_4_ using a glass pipette. Cartridges were washed with 1000 μL of 2% FA in H_2_O solution, followed by 1000 μL of 0.2% FA in MeOH solution. Analytes were then eluted into glass tubes using 600 μL of 2% ammonium hydroxide in MeOH solution. Eluates were evaporated to dryness under a stream of nitrogen. Finally, samples were resuspended in 250 μL of the resuspension solution (50 μL DMSO mixed with 200 μL of 94.5:2.5:2.5:0.5 ACN:MeOH:H_2_O:FA + 5 mM Amm. Form) and transferred into glass inserts fitted into 2 mL glass vials. A total of 10 μL of the sample was injected into the UPLC-TOF/MS system for analysis. A quality control (QC) sample was prepared by mixing together 20 μL of each sample from GD patients (*n* = 16) and healthy controls (*n* = 16) analyzed in the study.

### 3.6. Instrumentation and Parameters

The metabolomic study was performed using the Synapt G1 QTOF (Waters Corp., Milford, MA, USA) mass spectrometer coupled to an Acquity UPLC system (Waters Corp). Chromatographic separation of plasma components was achieved in a 45-min method using a HPLC Halo HILIC column (4.6 × 150 mm, 2.7 μm particle diameter) from Advanced Materials Technology (Wilmington, DE, USA), with a constant flow rate set at 0.5 mL/min and an operating temperature of 30 °C. Chromatographic mobile phase A was 94.5:2.5:2.5:0.5 ACN:MeOH:H_2_O:FA + 5 mM Amm. Form., while chromatographic mobile phase B was 10:89.5:0.5 ACN:H_2_O:FA + 5 mM Amm. Form. The “weak” and “strong” needle wash solutions were Phase A and 100% ACN, respectively. The gradient and in-depth parameters used to separate sample components are presented in Table 2.

The parameters for data acquisition, lock mass calibration, and MS-TOF are presented in Table 3. In order to maintain a high level of mass accuracy throughout the entire duration of the metabolomic run analysis, a standard solution of terfenadine (500 nM, ACN 5%, FA 0.2%), used for real-time calibration, was continuously infused throughout the analysis, using a lock mass probe connected to an isocratic pump model 515 (Waters Corp.). The flow rate of the isocratic pump was set at 1 mL/min. However, since a T splitter was used in the system, linking the pump to the lock mass probe, the actual flow rate of calibrant used was approximately 12 μL/min.

Following the UPLC-TOF/MS analysis, the peaks detected were processed using the software MarkerLynx XS (Waters Corp., Version 4.1) in order to align markers detected in samples based on their UPLC retention times and mass to charge ratios (*m/z*) and to evaluate their peak areas. Markers with a mass below 50 Da and above 1000 Da were also excluded in this step. Based on data obtained from the QC samples analyzed periodically throughout the metabolomic run in alternance with GD patient samples and healthy control samples, the mass window and the retention time window were set at 0.05 Da and 0.20 min. The intensity threshold was set at 5 counts. Data from over 91,000 different peaks detected in the samples analyzed were then transferred to EZinfo, a software of Umetrics (Umeå, Sweden) integrated to the Extended Statistics Tools Box of MassLynx v4.1 (Waters Corp.), for multivariate statistical analysis in order to identify compounds expressed differently in the GD patients group compared to the healthy control group. The Unit Variance (UV) and Pareto scaling algorithms, with and without logarithmic transformation, were tested.

Ultimately, UV scaling with logarithmic transformation was selected, since all the most promising biomarkers highlighted with the other scaling methods were also detected with this algorithm. UV scaling is expressed by the equation UV = $\frac{x\;-\;\bar{x}}{SD}$, where x is the area under the curve for a marker in a specific sample, whereas $\bar{x}$ and SD are the average and the standard deviation of the areas under the curve for the same marker measured in all samples, respectively. UV scaling reduces the emphasis of more abundant compounds while increasing the contribution of less abundant compounds, making all metabolites similarly important regardless of their respective abundance [44]. Logarithmic transformation, on the other hand, is used in order the correct the heteroscedasticity observed in the analyses [44]. Once an optimal scaling method was selected, the system stability throughout the study was inspected by evaluating the distribution of QC sample replicate injections (*n* = 8) performed periodically during the metabolomic run in a principal component analysis (PCA) chart. Following PCA analysis, an orthogonal partial least-square discriminant analysis (OPLS-DA) was used to target compounds with differential expression in the two groups under investigation. Finally, an S-plot was used to select metabolites contributing the most to the discrimination between the GD patients’ samples and the healthy control samples during OPLS-DA. Based on the trend plot chart distributions, metabolites that were expressed differently in the two groups were selected and further investigated.

Fragmentation tests using UPLC-quadrupole time-of-flight (QTOF) mode were performed to structurally elucidate the compounds of interest. These compounds were isolated in the quadrupole and fragmented in the collision cell, and the produced fragments were analyzed using the TOF analyzer to obtain their accurate masses. Main source ionization parameters and UPLC parameters remained unchanged for this procedure, while lock mass sampling cone voltage was increase to 12 to obtain a good signal for terfenadine.

## 4. Conclusions

The current metabolomic study has successfully identified and characterized seven potential biomarkers in the plasma of untreated GD patients using rapid SPE purification followed by a UPLC-TOF/MS analysis. As previously mentioned, some were already identified but not characterized by fragmentation studies. In summary, out of the 91,197 markers observed in plasma samples of GD patients and age- and gender-matched controls, lyso-Gb_1_ and four related analogs (−28 Da, −2 Da, +14 Da, and +18 Da), SPC, and N-palmitoyl-O-phosphocholineserine were highlighted as relevant to the disease by multivariate statistical analyses, such as OPLS-DA and S-plot charts. The lyso-Gb_1_ analog +18 Da has never been described in the literature, while lyso-Gb_1_ analogs −28 Da, −2 Da, and +14 Da have never been characterized using chromatographic separation or mass spectrometry fragmentation tests.

Although lyso-Gb_1_, SPC, and N-palmitoyl-O-phosphocholineserine have previously been observed in GD plasma samples, the metabolomic study has demonstrated the importance of evaluating a group of potential biomarkers to achieve a high level of sensitivity for a potential GD diagnostic test. Indeed, patient ID 14 did not have significantly elevated plasma levels of lyso-Gb_1_ or its related analogs. However, he did have increased levels of N-palmitoyl-O-phosphocholineserine. Therefore, a potential diagnostic test based solely on lyso-Gb_1_ would have provided a false negative result for this latter patient. This emphasizes the importance of having a diagnostic test based on a profile of biomarkers to be analyzed rather than only one biomarker. Considering that two biomarkers are not specific for GD (SPC and N-palmitoyl-O-phosphocholineserine) since they can be detected in other LSDs, we strongly recommend that these seven biomarkers be part of a biomarker profile for GD diagnosis, thus improving the overall specificity. In fact, having biomarkers that are not specific for GD highlights the importance of performing the enzyme analysis or molecular testing to confirm the diagnosis. However, the suggestion to use a profile of biomarkers might help for the early detection of GD patients. Potential test results, where N-palmitoyl-O-phosphocholineserine and SPC are increased, while lyso-Gb_1_ and its related analogs are not detected, might lead to a false differential diagnosis. While biomarker quantitation analysis is often the first test performed for potential GD patients, a thorough enzyme activity testing and molecular genetics analyses are necessary to confirm the diagnosis. Future perspectives will involve the development of a novel validated method for the quantitation of these biomarkers using a tandem mass spectrometry approach to thoroughly investigate potential correlations between biomarker concentrations and age, as well as clinical manifestations in treated and untreated GD patients for additional insights into the pathophysiology of the disease.

## 5. Study Limitations

Study limitations include the small number of untreated GD patients, as well as a low number of different mutations.

## Figures and Tables

**Table 1:** Demographics, mutations, treatment, and signs and symptoms of GD patients. For bone marrow burden: lower limb (LL) and spine (S) score on 8. Düsseldorf Gaucher Score (DGS) score on 8. Non-applicable (N/A); not evaluated (N/E); (x) = sign or symptom observed in GD patient; (-) = sign or symptom not observed in GD patient; female (F); male (M). It should be noted that the samples sent from different hospitals had different evaluation tests and procedures for their respective patients. Moreover, certain tests, such as bone marrow burden, were not assessed for all patients.

Patient ID	1	2	3	4	5	6	7	8	9	10	11	12	13	14	15	16
Age	62	49	43	62	22	47	49	70	63	71	62	43	70	53	24	44
Gender	F	F	F	F	F	M	F	F	M	M	F	F	F	F	M	F
Age at diagnosis	28	40	36	16	N/E	12	40	42	19	21	3	36	N/E	44	13	27
Mutations	N370S N370S	N370S N370S	N370S L444P	N370S N370S	N370S W184	N370S L444P	N370S N370S	N370S L444P	N370S R120W	N370S W378G	L444P L444P	N370S N370S	N370S L444P	N370S R496H	N370S L444P	L444P 84GG
Treatment	No	No	No	No	No	No	No	No	No	No	No	No	No	No	No	No
Bone disease																
Bone pain	-	Mild	Moderate	Mild	-	Moderate	-	-	-	Mild	Moderate	Mild	Moderate	-	-	Mild
Bone crisis	-	-	-	-	-	Moderate	-	-	Moderate	-	-	-	-	-	-	-
Erlenmeyer flask deformity	x	-	-	-	-		-	-	-		-	x	x	-	-	x
Bone marrow infiltration	x	x	x	x	-	x	-	-	-	x	-	x	x	-	x	x
Osteopenia	-	-	-	-	-	x	-	-	x	x	x	-	x	x	-	-
Infarction	-	-	-	-	-	x	-	-	x	-	-	-	x	-	-	x
Avascular necrosis	-	-	-	-	-	x	-	-	-	-	-	-	-	-	-	-
New fractures	-	-	-	-	-	-	-	-	-	-	-	-	-	-	-	-
Lytic lesions	x	-	-	-	-	-	-	-	x	-	-	-	-	-	-	-
Decreased bone mineral density	-	-	-	-	-	x	-	-	-	x	-	-	-	x	-	-
Bone marrow burden	DGS 5	LL 5 S 6–7	N/E	LL 4 S 3	N/E	LL 4 S 3	N/E	LL 7 S 5	DGS 7	N/E	N/E	LL 4 S 5–6	DGS 7	LL 2 S 3	N/E	LL 7 S 7
Hematological and visceral manifestations																
Anemia	-	-	x	-	-	-	-	-	x	-	x	x	-	-	-	-
Hemoglobin (g/L)	125	125	118	140	127	149	122	132	103	140	110	113	123	117	124	127
Thrombocytopenia and coagulopathy	-	x	x	x	-	-	x	-	x	-	-	x	x	-	x	x
Platelet counts (/L)	156	87	65	95	123	197	117	144	63	182	185	82	130	254	103	71
Splenomegaly	Mild	Mild	Mild	-	-	-	x	N/A	Moderate	N/A	N/A	-	N/A	-	-	N/A
Hepatomegaly	-	Mild	Mild	Mild	-	Mild	-	Mild	Moderate	N/E	Mild	Moderate	Mild	Moderate	Severe	-
Splenectomized	-	-	-	-	-	-	-	x	-	x	x	-	x	-	-	x
Gammopathies and malignancy	-	-	-	-	-	-	-	-	-	x	-	-	-	-	-	-
Pulmonary disease						-	-									
Elevated arterial pressure	-	-	-	-	-	-	-	-	-	x	x	-	-	-	-	-
Severe pulmonary hypertension	-	-	-	-	-	-	-	-	-	-	-	-	-	-	-	-
Hepatopulmonary syndrome	-	-	-	-	-	-	-	-	-	-	-	-	-	-	-	-
Respiratory failure	-	-	-	-	-	-	-	-	-	-	-	-	-	-	-	-
Other	-	-	-	-	-	-	-	-	-	-	-	-	-	-	-	-

**Table 2:** Ultra-performance liquid chromatography (UPLC) parameters for the separation of the plasma samples analyzed during the metabolomic study.

Parameters	Description
Column	Halo HILIC 2.7
ID x Length	4.6 × 150 mm
Particle size	2.7 μm
Column temperature	30 °C
Weak Wash solvent	94.5:2.5:2.5:0.5 ACN:MeOH:H_2_O:FA + 5 mM Amm. Form.
Strong Wash solvent	ACN
Injection volume	10 μL
Injection mode	Partial Loop
Autosampler temperature	10 °C
Mobile phase A	94.5:2.5:2.5:0.5 ACN:MeOH:H_2_O:FA + 5 mM Amm. Form.
Mobile phase B	10:89.5:0.5 ACN:H_2_O:FA + 5 mM Amm. Form.
Flow rate	0.5 mL/min
Gradient (% mobile phase B)	0 to 5min: 0%
	5.0 to 20.0 min: 0–10% (Linear gradient)
	20.0 to 25.0 min: 10%
	25.0 to 35.0 min: 10–60% (Linear gradient)
	35.0 to 40.0 min: 60
	40.0 to 45.0 min: 0%

**Table 3:** Mass spectrometry (MS) parameters for the quantitation of compounds present in the plasma samples analyzed during the metabolomic study.

Parameters	Description
Scan Mode	MS-TOF
Ionization Mode	Electrospray Ionization
Polarity	Positive
Analyzer Mode	V
Dynamic Range	Extended
Capillary Voltage	1.4 kV
Sampling Cone Voltage	10 V
Extraction Cone Voltage	5.0 V
Source Temperature	150 °C
Desolvation Temperature	450 °C
Cone Gas Flow	30 L/h
Desolvation Gas Flow	700 L/h
Trap Collision Energy	4.0 V
Transfer Collision Energy	2.0 V
Data Format	Centroid
Mass Range	50–1000 Da
Scan Time	0.1 s
**Lock mass**	
Compound	Terfenadine (500 nM)
Exact Mass	472.3215 Da
Solvent	94.5:5:0.5 H_2_O:ACN:FA
Scan Time	0.5 s
Interval	5.0 s
Sampling Cone Voltage	5 V
Trap Collision Energy	10
Mass Window	±0.2 Da
Scan Average	3
